# Incorporation of Elastin to Improve Polycaprolactone-Based Scaffolds for Skeletal Muscle via Electrospinning

**DOI:** 10.3390/polym13091501

**Published:** 2021-05-06

**Authors:** Victor Perez-Puyana, Paula Villanueva, Mercedes Jiménez-Rosado, Fernando de la Portilla, Alberto Romero

**Affiliations:** 1 Departamento de Ingeniería Química, Facultad de Química, Universidad de Sevilla, 41012 Sevilla, Spain; vperez11@us.es (V.P.-P.); alromero@us.es (A.R.); 2 Institute of Biomedicine of Seville (IBiS), “Virgen del Rocío” University Hospital, IBiS, CSIC/University of Seville, 41013 Sevilla, Spain; paulavg1995@gmail.com (P.V.); fportilla@us.es (F.d.l.P.)

**Keywords:** elastin, electrospinning, scaffolds, skeletal muscle cells

## Abstract

Skeletal muscle regeneration is increasingly necessary, which is reflected in the increasing number of studies that are focused on improving the scaffolds used for such regeneration, as well as the incubation protocol. The main objective of this work was to improve the characteristics of polycaprolactone (PCL) scaffolds by incorporating elastin to achieve better cell proliferation and biocompatibility. In addition, two cell incubation protocols (with and without dynamic mechanical stimulation) were evaluated to improve the activity and functionality yields of the regenerated cells. The results indicate that the incorporation of elastin generates aligned and more hydrophilic scaffolds with smaller fiber size. In addition, the mechanical properties of the resulting scaffolds make them adequate for use in both bioreactors and patients. All these characteristics increase the biocompatibility of these systems, generating a better interconnection with the tissue. However, due to the low maturation achieved in biological tests, no differences could be found between the incubation with and without dynamic mechanical stimulation.

## 1. Introduction

Regenerative medicine and specifically tissue engineering have grown exponentially in the number of publications, with the latter being one of the advances in biomedicine with greater impact [1,2]. This growth is due to the creation of new processing protocols that improve the quality of biomaterials [3]. For example, Kobayashi et al. proposed a standard protocol to prepare platelet-rich fibrin membranes [4], Zuidema et al. standardized the protocol for the correct rheological characterization of hydrogels for tissue engineering [5], and Xing et al. evaluated a quantity-controllable protocol for constructing individual tissue-engineered grafts [6].

In this field, skeletal muscle regeneration or substitution has increased its interest due to the large number of accidents and diseases that cause loss of muscle or muscle mass [7]. However, the techniques proposed to date are limited, since they not allow restoring the full functionality of the replaced muscle [8]. The failure of these techniques is usually due to the fact that the muscle cells are incubated ex vivo in a static manner, without undergoing the mechanical stimulation to which they are accustomed within the human body. This inactivity causes necrotic areas in the regenerated muscle and cells, which do not fulfill their functionality once settled in the patient [9]. Therefore, new protocols are being investigated, where muscle cells are dynamically stimulated during their incubation, improving their proliferation and differentiation. This is achieved using bioreactors that subject the cells to mechanical stimulation, recreating the conditions of muscle tissue in vivo [10,11]. However, this regeneration cannot be carried out directly on loose cells; therefore, a scaffold is necessary to support them. In this way, the cells are introduced into the scaffolds, which support the mechanical stimulation of the bioreactor and transfer it to the cells [12]. Thus, scaffolds must have the mechanical properties requested by the bioreactor, in addition to morphological properties suitable for the interconnection and proliferation of cells [13]. The biodegradability of the scaffolds is also important, since it must disappear once the muscle cells can fulfil their functionalities by themselves. Therefore, the development of a biomaterial and, specifically in our case, a scaffold for tissue engineering, is not based solely on the study of its manufacturing process. A complete evaluation of the scaffold must be carried out according to the type and duration of its contact with human tissue. In general, it is necessary to make a chemical and compositional characterization of the material. Moreover, biocompatibility must also be evaluated. Therefore, a biological evaluation of the material is required. These biological tests are based on in vitro and in vivo test methods, together with animal models. Such tests provide a good approximation for evaluating the behaviour of these materials [14].

Regarding the raw materials used to make the scaffolds, different polymers have been investigated [15]. For example, Riboldi et al. utilized polyester urethane membranes [16] and Patricio et al. characterize different polycaprolactone (PCL)/polylactic acid (PLA) scaffolds [17]. Among them, PCL has great potential due to its mechanical and morphological characteristics [18,19], although its surface properties, such as contact angle, do not confer good cell viability, unless it is combined with another biopolymer, such as alginate [20,21], collagen [22,23] or chitosan [24].

As a further innovation, for our study, dynamic stimulation provided by a bioreactor was added to this attempt to produce skeletal muscle in vitro. In addition, a polymeric combination of PCL/elastin-based scaffolds fabricated by electrospinning was used as support for muscle cells. Therefore, this work proposes the development of polymer-based scaffolds using a hybrid system developed from PCL and elastin via electrospinning. The approach adopted is the combination of PCL with elastin in order to tune the properties of PCL fibers to make them suitable for tissue engineering applications. To achieve this objective, a physicochemical, morphological and mechanical evaluation of the hybrid scaffolds was performed. In addition, a biological assessment through in vitro and in vivo studies was also carried out.

## 2. Materials and Methods

### 2.1. Materials

Elastin protein from bovine neck ligament was used as natural polymer (Sigma Aldrich, Steinheim, Germany). On the other hand, the synthetic polymer selected was poly(ε-caprolactone) (PCL) with a molecular weight of 45,000 g/mol. The solvent chosen for the hybrid solutions was 1,1,1,3,3,3-hexafluoro-2-propanol (HFIP). Both reagents were also purchased from Sigma Aldrich.

### 2.2. Electrospining Process

First of all, a polymer solution with PCL and elastin was prepared using 16 and 4 *w*/*v*%, respectively. These concentrations were selected because in previous studies it was demonstrated that a 4:1 ratio of PCL:elastin is the most optimal [25]. The solution was produced with HFIP as solvent at room temperature by stirring for ca. 24 h using a magnetic stirrer, before the electrospinning process. Once the solution was prepared, the electrospinning process was carried out in vertical mode with the following conditions: 14 kV (voltage), 0.4 mL/h (flow rate), 14 cm (needle-collector distance), 25 °C and 40% (temperature and humidity, respectively). The syringe used was a 10 mL syringe (with an 18G stainless steel needle). It was mentioning that the parameters used for the processing of PCL membranes utilized for comparison are similar.

### 2.3. Characterization of Nanofibrous Scaffolds

#### 2.3.1. Morphological Evaluation

The microscopy examination of the scaffolds was assessed with a XL 30 instrument (XL Series, Philips, Amsterdam, The Netherlands) at 15 kV. The samples were previously covered with a nanofilm of Au in a high-resolution sputter coater to improve the quality of the micrographs. A free digital processing software package, ImageJ, was used to determine the membrane porosity as well as the size of the fibers. Furthermore, the atomic composition of the scaffolds was examined with the energy dispersive spectroscopy capability (EDAX) of the scanning electron microscopy (SEM) equipment using an EDAX Si(Li) detector at an acceleration voltage of 5 kV.

#### 2.3.2. Physicochemical Evaluation

A physicochemical characterization was carried out with an iS50 ATR-FTIR spectrophotometer (Nicolet, Waltham, MA, USA). The different spectra were collected in the range of 4000–500 cm^−1^. In addition, the scaffolds’ wettability and hydrophobicity were assessed by water contact angle (WCA) measurements using a Drop Shape Analyser (Krüss, Hamburg, Germany). Both WCA values of the right and left sides of water droplets (volume of 5 µL approximately) were measured and the average value was calculated.

#### 2.3.3. Mechanical Evaluation

Tensile tests were performed using an ElectroForce 3200 (TA Instruments, New Castle, DE, USA), evaluating the evolution of the tensile load with the applied strain. The extensional rate was 0.1 mm s^−1^ at 20 °C. From the different measurements, three parameters were obtained: maximum stress, strain at break and Young’s modulus.

#### 2.3.4. Biological Evaluation

The scaffolds were biologically evaluated to assess cell behaviour. The cells used were rat skeletal myoblasts obtained from *Rattus norvegicus* L6 cell line (ATCC^®^ CRL-1458™) and were cultured in an incubator at 37 °C in the presence of 5% CO_2_. The growth medium used was Minimum Essential Medium α (12571-063, Gibco, Waltham, MA, USA) supplemented with 10% fetal bovine serum (FBS, Sigma) and 1% penicillin-streptomycin (P/S, 15140-122, Gibco). After the cells reached 85–90% of confluence, they were sub-cultured using trypsin-EDTA at 0.05% (25300-062, Gibco) and 20 × 10^6^ cells were seeded in each scaffold with growth medium. The scaffolds were cultured in a TC3 bioreactor (EBERS *Medical Technology* SL, Zaragoza, Spain) to be mechanically stimulated, inside the incubator, for 14 days (called dynamic scaffolds). After 7 days, the culture medium was changed to a differentiating medium of DMEM/high glucose with FBS at 2% and P/S at 1%. Static scaffolds (without mechanical stimulation) were used as control.

Every 72 h, a viability test (in vitro evaluation) was performed using Presto Blue (PrestoBlue™ Cell Viability Reagent, Invitrogen, Waltham, MA, USA) due to the presence of resazurin, a cell viability indicator, in its formulation.

Fourteen days after the beginning of the experiment, the scaffolds were extracted from the incubator and inserted in the animal model. The in vivo study encompassed 10 Wistar rats, in which two scaffolds (a dynamic scaffold and a static scaffold) were inserted and extracted after 30 days.

The scaffolds were then processed and stained with hematoxylin-eosin (H&E) (GHS316 Hematoxylin Solution, Gill No. 3; HT110116 500 ML: Eosin Y Solution, Sigma). The histological analysis was performed following Knightly’s classification [26], considering the reaction as slight (1 point, <25%), moderate (2 points, 25–75%) or severe (>75%). The variables evaluated were: acute inflammation, chronic inflammation, collagen deposition, fibroblast activity and neovascularization. In addition, the scaffolds were immunohistochemically stained with myogenin (Ab1835, Abcam, Cambridge, UK) to detect the degree of cell differentiation. The microscopy images were taken with the BX-61 microscope (Olympus, Tokyo, Japon) at 20×.

### 2.4. Ethical Considerations

This project is in the category of the Institutional Animal Care and Use Committee (IACUC) classification, since it includes procedures that cause or induce moderate pain, stress or discomfort, which are inhibited or eliminated with the required analgesics or anesthetics [27].

During the experimental period of this research project, the animals were treated according to the Council of Europe agreements for the protection of the animal experimental models used (Directive of the Council of Europe 86/609/EEC). In the different work procedures, the replacement and reduction of the number of animals was guaranteed, in addition to their housing, care and use. The pain, suffering and stress that these animals could potentially develop was minimized as much as possible.

The justification for the use of this experimental model is based on the absence of another type of procedure that allows achieving the expected results.

### 2.5. Statistical Analysis

At least three replicates were carried out for each measurement. Statistical analyses were performed with t tests and one-way analysis of variance (*p* < 0.05) using PASW Statistics for Windows (Version 18: SPSS, Chicago, IL, USA). Standard deviations were calculated for selected parameters.

## 3. Results & Discussion

### 3.1. Morphological Evaluation of Nanofibrous Scaffolds

**Figure 1:**
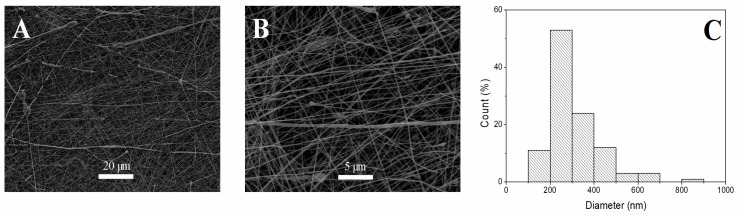
SEM images of PCL/elastin scaffolds at different magnifications: (**A**) 1000× and (**B**) 4000×. The fiber size distribution was also included (**C**).

Figure 1 shows the SEM images of electrospun mats obtained from the combination of PCL and elastin at different magnifications. Figure 1A shows a general overview of the microstructure of the obtained scaffold, whereas Figure 1B shows a better view of the fibers. In these micrographs, homogeneous fibers can be observed. Furthermore, Figure 1 also shows the fiber size distribution of the studied system (Figure 1C). The scaffold had a Gaussian distribution with respect to a central value (ranging between 200 and 300 nm). Thus, the PCL-elastin scaffold showed a homogeneous distribution towards that central value, with a mean fiber diameter of 269 nm (Table 1). This mean fiber diameter is slightly lower than the values obtained in other studies [28,29]. It is interesting to point out that smaller fiber sizes are more suitable to obtain a larger surface for cell adhesion [30]. In this way, PCL-elastin scaffolds could improve the adhesion achieved by PCL scaffolds with a larger fiber diameter (451 nm). In addition to fiber size, the alignment of the fibers was also calculated, showing a relatively aligned structure with a general alignment of 50% (Table 1). This slight alignment could be beneficial for the growth and interconnection of cells in a given orientation, which is interesting for some muscles, such as myotubes [31] or endothelium [32].

Furthermore, the presence of protein in the network of the fibrous membrane can be identified by the presence of nitrogen in it. Thus, an EDAX analysis was performed with the SEM images to confirm that the electrospun fibers contained proteins in their structure. The nitrogen present in the surface obtained from the EDAX profile is shown in Table 1, with an average value of 2.51%. This amount of protein contributes to energy barriers that must be overcome during cell adhesion, improving the biocompatibility of scaffolds [33].

### 3.2. Physicochemical Evaluation of Nanofibrous Scaffolds

**Figure 2:**
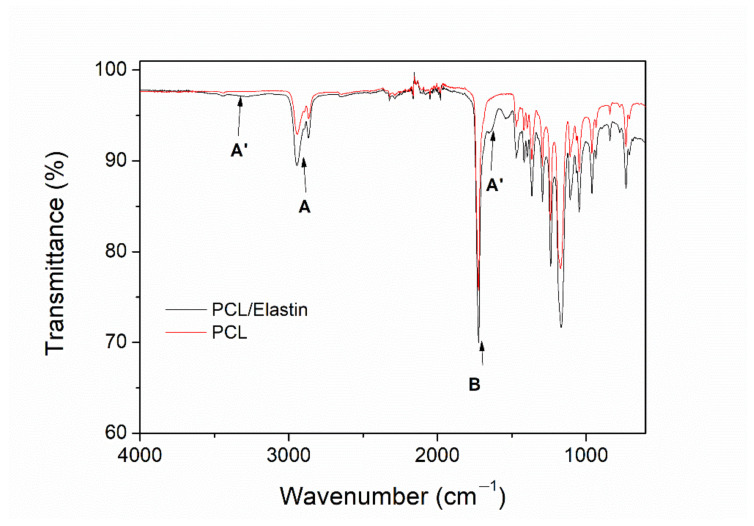
FTIR profile of PCL/elastin and PCL scaffolds.

The presence of protein in the structure of the scaffold can also be analyzed from the FTIR profile (Figure 2). The spectrum presented a profile with the characteristic peaks of PCL, together with the typical bands of proteins. PCL is responsible for two important areas: bands at 2950 and 2860 cm^−1^ (A) related to the CH_2_ symmetrical and asymmetrical stretching and a sharp band that appears at 1725 cm^−1^ (B), associated with carbonyl stretching [34]. In addition to this, the bands from elastin (protein) are: a broad area at ca. 3280 cm^−1^ (A′) associated with N-H stretching (amide A signal), attenuated due to the low concentration of protein compared to PCL, and bands at 1635 and 1525 cm^−1^ (B′) related to carbonyl stretching and C-N stretching of amides, respectively [35,36].

The wettability of the obtained scaffolds was also measured, as is observed in Table 1, in order to study their hydrophobicity. The hybrid system presented a contact angle of *ca* 68°, much lower than the value of pure PCL systems, which are in the range of 90–100° [37]. This value allows determining that the scaffold is hydrophilic according to the studies of Kubiak and Mathia [38]. This hydrophilic character could be due to the presence of elastin on the surface of the fibers, which is much more hydrophilic than PCL. The hydrophilicity acquired due to the inclusion of elastin (protein) is suitable for cell adhesion [39]. According to the obtained value, it can be confirmed that the presence of elastin produced a decrease in the contact angle and, therefore, a more hydrophilic system that could improve its cell adhesion and proliferation. This behaviour is due to the influence of the protein on the energy barriers, as was previously commented.

### 3.3. Mechanical Characterization of Nanofibrous Scaffolds

The analysis of the different parameters obtained from the strain-stress curves is shown in Table 1. All the systems showed a similar behaviour, with a linear increase in the strain/stress profile until a maximum value is reached, since when a slight decrease of the slope take place until the sample is broken, with the subsequent decrease in the profile (profile not shown). According to the results shown, the decrease of the fiber size showed in Section 3.1 is not correlated with the obtained mechanical properties. As a general fact, the addition of elastin to PCL scaffolds produced a reinforcement of the structure, based on the increase in both Young’s modulus and maximum stress with respect to the PCL reference system. However, the produced system is less deformable, as is shown by the decrease in the strain at break, compared to the values obtained for pure PCL scaffolds [37].

### 3.4. Biological Evaluation of Nanofibrous Scaffolds

#### 3.4.1. “In Vitro” Evaluation

**Figure 3:**
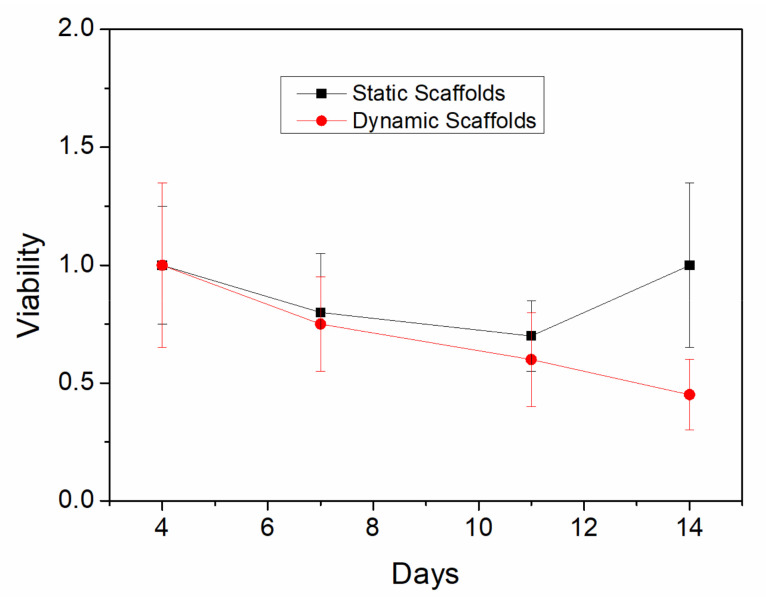
Cell viability of the PCL/elastin scaffolds after a static and dynamic protocol in a bioreactor.

The evolution of cell viability during the 14 days of incubation in non-stimulated (static) and stimulated (dynamic) scaffolds is shown in Figure 3. 

Both groups showed a decrease in viability at 7 and 11 days of culture. However, on the last day of culture, viability growth was observed for the static controls, while in the dynamic scaffolds the viability continued to decrease. These results suggest that cells may have detached from the dynamic scaffold during their culture in the bioreactor to a greater extent compared to the static controls, due to the continuous tension and contraction movements, which may influence the feasibility results. The statistical test performed between the groups was the Mann-Whitney test, which revealed only significant differences between the groups on day 14. Nevertheless, it is worth mentioning that PCL-elastin scaffolds always allow greater cell viability than PCL systems, where viability is around 0.3–0.5 after 4–7 days [40,41].

#### 3.4.2. “In Vivo” Evaluation

##### H&E Staining

During the histological study, the variables described in Section 2.3.4 were analyzed (Table 2). In most cases there was a slightly acute inflammatory process (80%), with more cases of higher and chronic inflammation in the static controls. Fibroblastic proliferation and collagen formations were also mostly slight and somewhat higher in the static controls. In addition, foci of neovascularization were found, especially in dynamic scaffolds, which may lead to think that, with a longer period of implantation, these foci may be larger. In all cases, the inflammation caused was similar to that produced by PCL systems, although fibroblastic proliferation and collagen formations indicate that the incorporation of elastin in the scaffolds improves the patient’s reaction to them, not generating excessive encapsulation of the biomaterial, which may cause a malfunction of the scaffold [41].

On the one hand, the static controls presented 20% of moderate-severe acute inflammation, increasing this figure in chronic inflammation to 70%. Moderate-severe fibrosis was present in half of the scaffolds and only 30% had moderate neovascularization. On the other hand, the dynamic scaffolds presented only 20% of moderate-severe acute inflammation, increasing in chronic inflammation to 40%. Fibrosis was slight in 70% of the cases, and half of the scaffolds had moderate-severe neovascularization.

**Figure 4:**
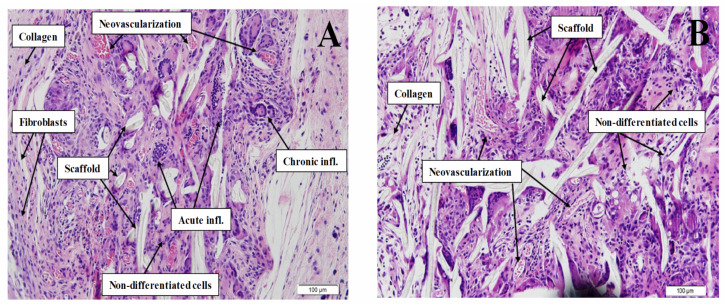
H&E photomicrographs (magnification of 2×) of the (**A**) static and (**B**) dynamic PCL/elastin scaffolds seeded with cells. Different aspects were shown with arrows: collagen formation, neovascularization, undifferentiated cells, inflammations and marks of the presence of the scaffold.

Based on Figure 4, the assessment of skeletal muscle creation reveals that, although the static samples (Figure 4A) and the dynamic samples (Figure 4B) show good cell viability, the cells observed in both groups presented incomplete differentiation, that is, they were not muscle fibers.

##### Immunohistochemistry

**Figure 5:**
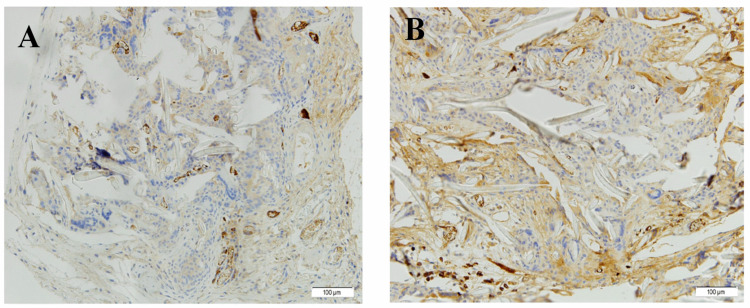
SMA antibody immunohistochemistry (magnification of 20×) of the (**A**) static and (**B**) dynamic PCL/elastin scaffolds seeded with cells.

Regarding the immunohistochemical study, the images shown in Figure 5 reveal the presence of stained nuclei due to their differentiation. However, for both static samples (Figure 5A) and dynamic samples (Figure 5B), the microscopic assessment reveals that differentiation had not been complete due to incomplete maturation. No pathological differences were found between the static controls and the implanted dynamic scaffolds.

## 4. Conclusions

Hybrid nanofibrous PCL/elastin scaffolds with a huge potential for their application in tissue engineering were obtained by electrospinning. The addition of elastin to PCL-based scaffolds produced more hydrophilic scaffolds with a smaller mean fiber size, although with higher Young’s modulus and maximum stress. In contrast, the formed structures were less deformable due to their lower strain at break. All this caused the incorporation of elastin in the systems to improve their biocompatibility.

According to the biological results, the in vitro creation of muscle tissue in an immature phase was shown. An increase in viability in the static controls versus the dynamic scaffolds was observed during the last phase of in vitro culture. Among in vivo analyses, fibroblast activity and collagen deposition were similar in both groups, since the materials used in both types of scaffolds were identical. Lastly, neovascularization was greater in dynamic scaffolds due to the positive effect of dynamic stimulation in the bioreactor, which is an encouraging result to continue working with these systems.

Further studies will encompass the development of the in vitro experimental phase to obtain more mature muscle tissue prior to implantation. To this end, the next step will be to optimize the dynamic stimulation protocol of the bioreactor, increasing the culture time, adding pauses to the stimulation and optimizing the stimulation speed. All this can improve their implementation as related biomaterials for the regeneration of muscular tissue in digestive applications (i.e., intestine, stomach or esophagus).

## Figures and Tables

**Table 1:** Mean fiber diameter, alignment, protein content, contact angle, Young’s modulus, strain at break and maximum stress values of PCL/elastin scaffolds. Some of the parameters obtained for PCL scaffolds were also included as reference. * means that there is no data reported

System	Fiber Diameter (nm)	Alignment (%)	Protein Content (%)	Contact Angle (°)	Young’s Modulus (MPa)	Strain at Break (%)	Maximum Stress (MPa)
PCL-Elastin	269 ± 84	50 ± 7	2.5 ± 0.5	67.5 ± 3.2	120.0 ± 28.5	25.3 ± 4.2	18.1 ± 3.9
PCL	451 ± 62	*	*	102 ± 11	35 ± 2.4	55 ± 6.7	10.6

* no data reported.

**Table 2:** Summary of the different parameters measured during the biological evaluation of the static and dynamic PCL/elastin scaffolds: acute inflammation, chronic inflammation, collagen deposition, fibroblast activity and neovascularization.

Parameters	Acute Inflammation		Chronic Inflammation		Collagen Deposition		Fibroblast ACTIVITY		Neovascularization	
Grade	Static Scaffolds	Dynamic Scaffolds	Static Scaffolds	Dynamic Scaffolds	Static Scaffolds	Dynamic Scaffolds	Static Scaffolds	Dynamic Scaffolds	Static Scaffolds	Dynamic Scaffolds
Slight	8	8	5	6	5	7	5	7	7	5
Moderate	1	2	4	3	4	1	5	1	3	3
Severe	1	0	1	1	1	2	0	2	0	2
TOTAL	10	10	10	10	10	10	10	10	10	10

## Data Availability

The data presented in this study are available on request from the corresponding author.
